# 3.E. Workshop: Is (Planetary) health a priority in international deals?

**DOI:** 10.1093/eurpub/ckac129.141

**Published:** 2022-10-25

**Authors:** Organised by: EUPHA-ENV, Chair persons: Marija Jevtic (EUPHA-ENV), Vlatka Matkovic (Belgium)

## Abstract

This workshop is dedicated to different types of deals/arrangements relating environmental health to planetary health, healthy energy, arrangements linking science to actions, and statement given by experts to recognise health in ambitious initiatives toward zero pollution. By presentations about planetary health education, it will underline the role of training as a cornerstone for international sustainable public health deals. The Lancet Public Health underlines: “No public health without planetary health”, the future health of the planet and human health are inextricably linked. Planetary health should be part of all global, national but also regional and local deals to improve sustainability worldwide. Further research and applications of the public health (PH) Education Framework are needed in order to increase evidence and awareness in PH and strengthen PH collaboration in Europe and beyond. Workshop will also discuss the European response and transformation towards healthy energy, as a part of many agreements/deals, especially European Green Deal. Science and the accelerating scale of environmental degradation tells us we have to be more ambitious and strive to zero pollution. Health-based approach can be the overarching principle to deliver on these ambitions. For a healthy planet for healthy people we need to have more determined action plans that will deliver on zero pollution and have a sound health-based approach. To achieve better public health, false solutions to the current energy, unsustainable and polluting energy crisis, is to go ambitiously for zero-pollution solutions. Looking for a strong argument for Clean air deals, the researchers will present their preliminary results of costs of air pollution impact on health in the Western Balkans (WB). It is well documented that poor air quality is responsible for increased risk of mortality and morbidity. The WB region was selected for this study because it is one of the air pollution hotspots in Europe where the levels of PM2.5, PM10, NO2 and O3 are frequently above the EU Air Quality Directive guidelines. Air pollution impact on health has a relatively higher economic burden in the WB compared to EU27 due to both the higher pollution levels and the lower per capita GDP. The external costs of air pollution is a strong argument to consider health as a priority in every policy area at national level as well as international agreements. Furthermore workshop will also present an initiative regarding health experts’ voice for Healthier Choice - a call for Zero Air Pollution in the WB. Health experts in the WB have joined forces to highlight the importance of air quality actions and achieving zero air pollution as a prevention intervention for public health. Health experts highlight that in Green Agenda for the WB the most needed are clear goals of achieving zero pollution and a timeline in the near future.

**Key messages:**

• Planetary health should be part of all global, national but also regional and local deals to improve sustainability worldwide.

• The external costs of air pollution is a strong argument to consider health as a priority in every policy area at national level as well as international agreements.

